# Long Noncoding RNA LINC00460 Promotes the Gefitinib Resistance of Nonsmall Cell Lung Cancer Through Epidermal Growth Factor Receptor by Sponging miR-769-5p

**DOI:** 10.1089/dna.2018.4462

**Published:** 2019-02-05

**Authors:** Guodong Ma, Jiping Zhu, Feng Liu, Yan Yang

**Affiliations:** ^1^ Department of Chest Surgery, Nanjing Chest Hospital, Nanjing, China.; ^2^ Department of Pneumology, Jiangsu Province Hospital of Traditional Chinese Medicine, Nanjing, China.; ^3^ Department of Radiation Oncology, The First Affiliated Hospital of Nanjing Medical University, Nanjing, China.

**Keywords:** nonsmall cell lung cancer, gefitinib resistance, LINC00460, miR-769-5p, EGFR

## Abstract

The vital roles of long noncoding RNAs (lncRNAs) in the nonsmall cell lung cancer (NSCLC) tumorigenesis are increasingly important. This work aims to investigate the role of lncRNA LINC00460 in the gefitinib resistance of NSCLC cells and discover its relevant mechanism. Our finding reveals that the expression of lncRNA LINC00460 is upregulated in the gefitinib-resistant NSCLC tissue and cells, and closely correlated with advanced tumor stage and clinical poor prognosis outcome. Gain and loss functional assays are performed in gefitinib-resistant NSCLC cells (A549/GR), stating that LINC00460 facilitates the 50% inhibitive concentration of gefitinib for NSCLC cells, multidrug-resistant-related proteins (P-gp, MRP1, and BCRP), as well as the invasion. *In vivo*, LINC00460 silencing represses the tumor growth. Bioinformatics prediction tools and luciferase analysis confirm that the upregulated LINC00460 sponged miR-769-5p in NSCLC cells; moreover, epidermal growth factor receptor (EGFR) is identified as a direct target gene of miR-769-5p. Verification experiments confirm that the restoration of EGFR could weaken the sensibility of NSCLC cells toward the gefitinib. In conclusion, our result demonstrates that LINC00460 plays a pivotal role in gefitinib resistance of NSCLC cells by targeting EGFR through sponging miR-769-5p. This finding might serve as a therapeutic target for NSCLC.

## Introduction

Nonsmall cell lung cancer (NSCLC) is one of the most common types of human cancers that cause large number of cancer-related deaths worldwide, accounting for 85% of all lung cancer (Hisakane *et al.*, 2017; Guo *et al.*, 2018; Shen *et al.*, 2018; Wu *et al.*, 2018). The long-term clinical therapeutic effects and survival rates of NSCLC patients have been negative, with as low as 15% of the 5-year survival rate (Anzai *et al.*, 2017; Chiang *et al.*, 2018). For a subtype of NSCLC patients who are marked with epidermal growth factor receptor (EGFR)-sensitive mutations, EGFR tyrosine kinase inhibitors (EGFR-TKIs) are developed to resist epithelial-derived solid tumor, such as gefitinib for advanced NSCLC (Peng *et al.*, 2016; Jiang *et al.*, 2017b; Li *et al.*, 2017). Gefitinib functions as a first-line single agent for NSCLC treatment. Unfortunately, the acquired drug resistance toward EGFR-TKIs appears with high frequency and leads to a limitation of gefitinib in the clinical treatment (Jiang *et al.*, 2017a).

Noncoding RNAs (ncRNAs) are groups of transcripts without the protein-coding potential (Hu *et al.*, 2017). ncRNAs contain two categories: long noncoding RNAs (lncRNAs) with >200 nucleotides and microRNAs (miRNAs) with 20–23 nucleotides (Zhu *et al.*, 2017). The genes that code lncRNAs are characterized by positional conservation and short stretches within species (Zhang *et al.*, 2018). Up to now, the vital roles of lncRNAs have significantly developed, including transcription regulation and post-transcription regulation at cellular levels (Zhu *et al.*, 2017). For example, in the NSCLC, the LINC00339 promoted the progression through FOXM1 by targeting miR-145, showing the important role of the LINC00339/miR-145/FOXM1 axis in the NSCLC tumorigenesis (Yuan *et al.*, 2018).

The gene mutations cause the resistance to TKIs in patients with NSCLC. Moreover, the structural analysis of binding modes of gefitinib with EGFR is investigated (Liu and Gray, 2006). Bello (2018) performed structural analysis, and showed that the drugs impact differently the conformational space of active and inactive EGFRs, and some ligands have better affinity for the inactive EGFR than for the active EGFR state. Energetic analysis shows that lapatinib and TAK-285 have better affinity for inactive EGFR than for the active EGFR state or HER. Besides, new potential inhibitors are designed and screened *in silico* (Ahmed *et al.*, 2013; Awasthi *et al.*, 2014).

In this study, our data demonstrated that LINC00460 is significantly upregulated in gefitinib-resistant NSCLC cells compared with the sensitive cells. Besides, LINC00460 is highly regulated in the gefitinib-resistant NSCLC cells. More interestingly, LINC00460 promotes the gefitinib resistance of NSCLC cells by sponging miR-769-5p, thereby promoting EGFR expression. This finding could provide a new insight for the NSCLC therapies.

## Materials and Methods

### Ethics statement and tissue samples

All the clinical recruitment and experimentation were approved by the Ethics Committee of the First Affiliated Hospital of Nanjing Medical University (Ethical approval number: J201600822). This study enrolled 36 patients who underwent surgical section and were pathologically diagnosed with NSCLC by pathologists. The clinical and clinicopathological data were recorded in detail. All enrolled patients signed the informed consent and were in agreement.

### NSCLC cells and culture

NSCLC cell lines (H460, A549, SK-MES-1, and H1299) and normal human bronchial epithelial cells were both purchased by the ATCC (American Type Culture Collection). Cells were cultured in RPMI-1640 medium (Gibco; Thermo Fisher Scientific, Inc., Waltham, MA), supplemented with fetal bovine serum (FBS, 10%; Gibco BRL, NY) at 37°C in humidified atmosphere with 5% CO_2_.

### Cells' transfection

A549 and A549/GR cells were transfected with the silencing siRNA and overexpression plasmid (pcDNA3.1) for LINC00460 (pcDNA-LINC00460 and siRNA-LINC00460). Oligonucleotides were designed and synthesized by Santa Cruz Biotechnology, Inc. (Dallas, TX) and transfected (50 nM) using Lipofectamine 2000 (Invitrogen, Carlsbad, CA) according to the manufacturer's instructions. Sequences are shown in Supplementary Table S1.

### RNA extraction and quantitative PCR

As previously described, RNA extraction was performed using TRIzol (Invitrogen ). Then, the cDNA was synthesized, and quantitative PCR (qRT-PCR) was carried out using reaction system. The relative expression level of the RNA was calculated using the 2^−ΔΔCt^ methods. Glyceraldehyde 3-phosphate dehydrogenase (GAPDH) was used as the endogenous control. The sequences of the primers used for qRT-PCR analysis are listed in Supplementary Table S1.

### Cell counting kit-8 assay for drug resistance

The drug resistance was measured using cell counting kit-8 (CCK8) assays. A549 and A549/GR cells were transfected with oligonucleotides or plasmid, and then treated with gefitinib (0–20 μM). After 24 h, the 10 μL of CCK-8 reagent was added and incubated, and the absorbance was measured at 450 nm. The blank transfection was set as normalization 100% survival. The 50% maximal inhibitory concentration (IC50) was measured for each chemotherapeutic drug.

### Cell invasion assays

The invasion assay was performed using matrigel invasion chambers (BD Biosciences, SanJose, CA) according to the manufacturer's instructions. In brief, cells were seeded on the upper floor of the chambers. The lower floor was added with medium containing 10% FBS. After 1 day, the cells number that invaded through member were fixed with 4% paraformaldehyde and stained with 0.5% crystal violet. Among five randomly selected views, the number of invasive cells was counted.

### Cell cytoplasm/nucleus fraction isolation

The cellular fragments were extracted using Nuclear and Cytoplasmic Extraction Reagents (Thermo Scientific). The subcellular location of LINC00460 was tested using real-time polymerase chain reaction (RT-PCR) assays.

### Western blotting

Cells were treated with radio-immunoprecipitation assay lysis reagent (Millipore, Billerica, MA), and the lysates were prepared with phosphatase inhibitors and protease inhibitors (Roche, Basel, Switzerland). BCA Protein Assay Kit (Thermo Fisher) was used to determine the protein concentration. Primary antibodies were purchased from Abcam Company, including anti-EGFR (1:1000 dilution) and anti-GAPDH (1:1000 dilution). Blots were detected using Immobilon-Western Chemiluminescent Kit (Millipore) and visualized by ImageJ software.

### Luciferase reporter assay

The 3′-UTR luciferase reporter containing the LINC00460 and EGFR matching with miR-769-5p was constructed, as well as the site deletion controls. A549 cells at a density of 5 × 10^4^ cells/well were transfected with the luciferase reporter and/or miR-769-5p. Luciferase activity was analyzed and normalized to Renilla luciferase transfection.

### Tumor xenograft *in vivo* model

The animal study was approved by the Animal Ethics Committee of The First Affiliated Hospital of Nanjing Medical University. Male nude mice (10 mice, 4-week old) were purchased from the Academy of Military Medical Science (Beijing, China). Cells were resuspended in PBS and injected into the flank of mice (5 × 10^6^ cells).

### Statistical analyses

The data of each assay was analyzed and presented as mean ± SD from repeat three independent experiments. The statistical significance was analyzed by two-tailed Student's *t*-test and/or one-way analysis of variance using SPSS 20.0 software.

## Results

### lncRNA LINC00460 presents the highly expressed levels in the NSCLC tissue and indicates the unfavorable prognosis

**FIG. 1.**
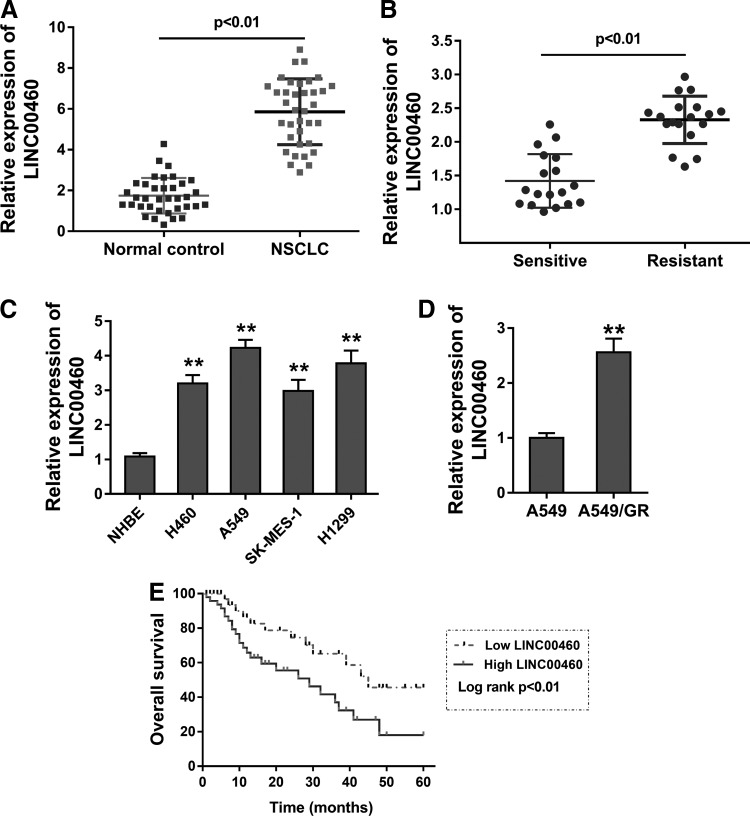
lncRNA LINC00460 presents the highly expressed levels in the NSCLC tissue and unfavorable prognosis. **(A)** RT-PCR analysis revealed the levels of LINC00460 in clinically enrolled NSCLC tissue. **(B)** RT-PCR analysis revealed the lncRNA LINC00460 expression in the NSCLC patients who were diagnosed with gefitinib resistance, or sensitive to the gefitinib chemotherapy. **(C)** LINC00460 expression in the NSCLC cell lines compared with normal cells. **(D)** LINC00460 expression in gefitinib-resistant cells (A549/GR, gefitinib resistant) and cultured parental A549 cells. **(E)** Survival analysis revealed the survival rate of NSCLC patients who were accompanied by high LINC00460 expression or lower individuals. Data are expressed as mean ± SD. **p < 0.01 represents statistical difference. lncRNA, long noncoding RNA; NSCLC, nonsmall cell lung cancer; RT-PCR, real-time polymerase chain reaction.

It has been reported that LINC00460 is found to be overexpressed in human cancer, such as meningioma and laryngeal squamous cell carcinoma. To investigate the unknown roles of LINC00460 in NSCLC, we performed the RT-PCR analysis to measure the levels of LINC00460 in clinically enrolled NSCLC tissue (Table 1). Data stated that LINC00460 expression was highly observed in the NSCLC samples compared with the normal adjacent lung tissue (Fig. 1A). Furthermore, in the NSCLC patients who were diagnosed with gefitinib resistance, lncRNA LINC00460 expression was upregulated compared with those who were sensitive to the gefitinib chemotherapy (Fig. 1B). Then, in the NSCLC cell lines, LINC00460 expression levels were almost significantly increased compared with normal cells (Fig. 1C). After that, we cultured the gefitinib-resistant cells (A549/GR, Gefitinib Resistant). Results showed that LINC00460 expression was enhanced in the A549/GR than in the A549 cells (Fig. 1D). Finally, survival analysis revealed that these NSCLC who accompanied by high LINC00460 expression had unfavorable prognosis than the lower individuals (Fig. 1E). Thus, lncRNA LINC00460 presents the highly expressed levels in the NSCLC tissue and unfavorable prognosis.

**Table 1.** Relationship Between LINC00460 Expression and Clinicopathological Characteristics of Nonsmall Cell Lung Cancer Patients

		*LINC00460*		
	*Total = 36*	*Low (15)*	*High (21)*	p
Gender				
Male	26	9	17	0.648
Female	10	6	4	
Age				
≤60	19	8	11	0.402
>60	17	7	10	
Smoking				
Yes	16	6	10	0.725
No	20	9	11	
Size of tumor (cm)				
≥3	23	11	12	0.012^*^
<3	13	4	9	
Differentiation				
Well/moderate	24	10	14	0.684
Poor	12	5	7	
TNM stage				
I/II	20	8	12	0.010^*^
III/IV	16	7	9	
Lymph node metastasis				
N0	19	9	10	0.087
N1–3	17	6	11	

^*^ *p* < 0.05 represents statistical difference.

### LINC00460 accelerates the gefitinib chemotherapy resistance, invasion, and tumor growth in NSCLC cells

**FIG. 2.**
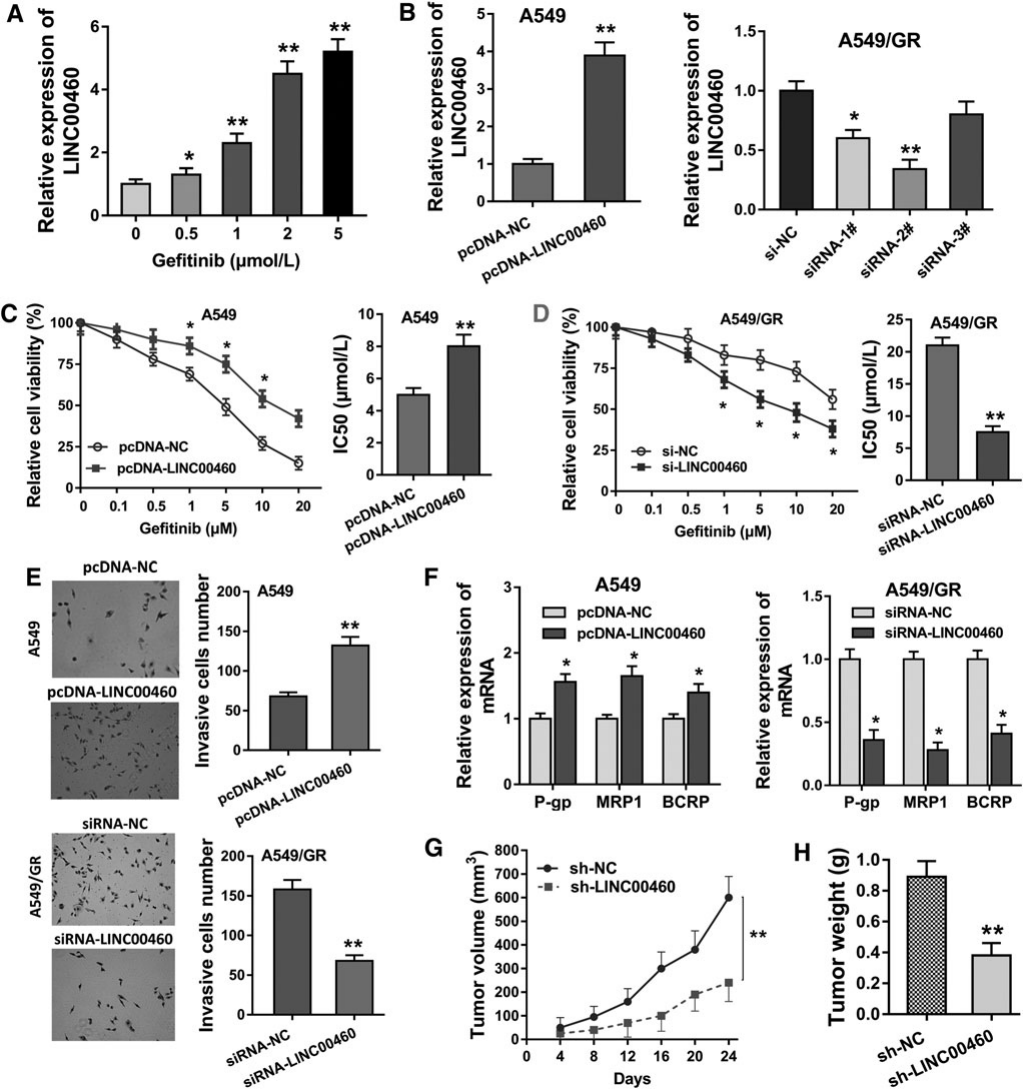
LINC00460 accelerates the gefitinib chemotherapy resistance, invasion, and tumor growth in NSCLC cells. **(A)** RT-PCR revealed the LINC00460 expression in NSCLC cells (A549) administered with increasing concentration of gefitinib. **(B)** A549 cells were transfected with LINC00460 oligonucleotides, and gefitinib-resistant A549 cells (A549/GR) were transfected with LINC00460 plasmids. **(C, D)** Chemotherapy-sensitive test by CCK-8 revealed the IC50 value for gefitinib in A549 cells and A549/GR cells. **(E)** Transwell assays revealed the invasive cell count in A549 cells and A549/GR cells. **(F)** Multidrug-resistant-related protein (P-gp, MRP1, and BCRP) expression levels were measured using RT-PCR in A549 cells and A549/GR cells. **(G, H)** Xenograft mice *in vivo* assay showed the tumor volume and weight in the mice injected with A549 cells. Data are expressed as mean ± SD. *p < 0.05, **p < 0.01 represents statistical difference. CCK-8, cell counting kit-8; IC50, 50% maximal inhibitory concentration.

It was discovered that LINC00460 expression was upregulated both in the NSCLC tissue and in the gefitinib-resistant tissue. Then, in the NSCLC cells (A549), LINC00460 expression was increasingly enhanced in the administration of increasing concentration of gefitinib (Fig. 2A). Gain and loss functional assays were conducted in the A549 cells and gefitinib-resistant A549 cells (A549/GR) through oligonucleotides or plasmids transfection (Fig. 2B). Chemotherapy-sensitive test using CCK-8 revealed that IC50 value for gefitinib was increased in A549 cells transfected with LINC00460 plasmids (Fig. 2C) or decreased in A549/GR cells transfected with siRNA-LINC00460 (Fig. 2D). Transwell assays revealed that enhanced LINC00460 expression accelerated the invasive cell count, and LINC00460 silencing inhibited the invasive cell count (Fig. 2E). RT-PCR analysis stated that enhanced LINC00460 expression accelerated the multidrug-resistant-related protein (P-gp, MRP1, and BCRP) expression, and LINC00460 silencing inhibited them (Fig. 2F). Xenograft mice *in vivo* assay showed that LINC00460 silencing suppressed the tumor volume and weight in the group injected with A549 cells (Fig. 2G, H). Overall, the cellular functional data demonstrated that LINC00460 accelerates the gefitinib chemotherapy resistance, invasion, and tumor growth in NSCLC cells.

### LINC00460 regulates the EGFR protein through sponging miR-769-5p

**FIG. 3.**
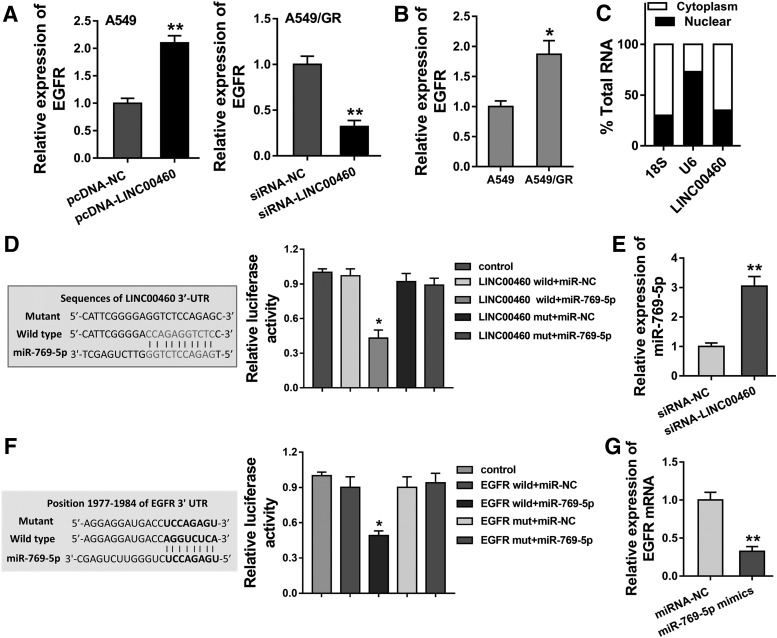
LINC00460 regulates the EGFR protein through sponging miR-769-5p. **(A)** EGFR mRNA expression was measured in the gefitinib chemotherapy resistance of NSCLC cells (A549/GR) and A549 cells transfected with siRNA and plasmids. **(B)** EGFR mRNA expression was measured in the gefitinib chemotherapy resistance of NSCLC cells (A549/GR) and A549 cells. **(C)** Subcellular fractionation analysis showed the distribution of LINC00460 in the cytoplasm. **(D)** Schematic diagram for the LINC00460 3′-UTR and miR-769-5p. Luciferase assay was performed to confirm it. **(E)** miR-769-5p expression was measured using PCR in the A549/GR cells transfected with siRNA-LINC00460. **(F)** Schematic diagram for the EGFR 3′-UTR and miR-769-5p. Luciferase assay was performed to confirm it. **(G)** EGFR mRNA expression was measured in A549/GR cells transfected with miR-769-5p mimics. Data are expressed as mean ± SD. *p < 0.05, **p < 0.01 represents statistical difference. EGFR, epidermal growth factor receptor.

To discover the in-depth mechanism that LINC00460 accelerates the gefitinib chemotherapy resistance, invasion, and tumor growth in NSCLC cells, we performed the following assays for mechanism research. We noticed that the upregulation or silencing of LINC00460 could increase or decrease the EGFR mRNA expression (Fig. 3A). Besides, the level of EGFR was upregulated in the gefitinib chemotherapy resistance of NSCLC cells (A549/GR) compared with control cells (Fig. 3B). This interesting finding sparks the inspiration whether LINC00460 positively regulates EGFR expression through post-transcriptional control. Subcellular fractionation analysis revealed the distribution of LINC00460 mainly in the cytoplasm (Fig. 3C). The evidence supported the potential of post-transcriptional regulation of LINC00460. Then, being helped by bioinformatics tool programs and luciferase assay, we confirmed that LINC00460 harbored the miR-769-5p as a miRNA “sponge” (Fig. 3D). Subsequently, we confirmed the binding within miR-769-5p and EGFR mRNA 3′-UTR using the same methods (Fig. 3F). Moreover, in NSCLC cells, the transfection of LINC00460 siRNA enhanced the miR-769-5p expression (Fig. 3E), and transfection of miR-769-5p mimics knocked down the EGFR mRNA level (Fig. 3G). In conclusion, we show that the LINC00460 regulates the EGFR protein through sponging miR-769-5p, constituting LINC00460-miR-769-5p-EGFR axis.

### EGFR enhances the role of LINC00460 in the gefitinib chemotherapy resistance of NSCLC cells

**FIG. 4.**
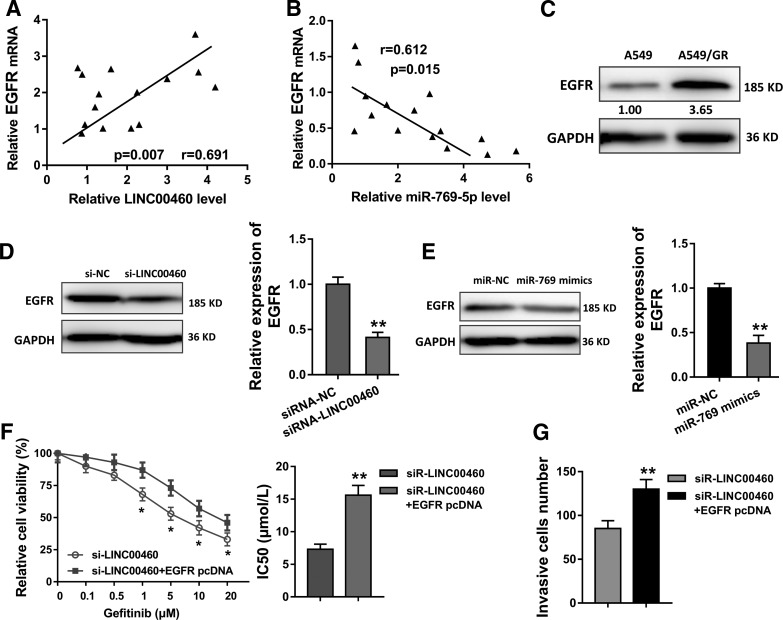
EGFR enhances the role of LINC00460 in the gefitinib chemotherapy resistance of NSCLC cells. **(A, B)** The correlation of EGFR with LINC00460 and miR-769-5p was analyzed using Pearson's correlation analysis. **(C)** Western blots showed the EGFR protein expression in control cells (A549) and gefitinib-resistant NSCLC cells (A549/GR). **(D, E)** EGFR protein expression in A549/GR cells transfected with si-LINC00460 and miR-769-5p mimics. **(F)** Chemotherapy-sensitive test with CCK-8 revealed the IC50 value of A549/GR cells cotransfected with the siRNA or/and EGFR overexpression plasmids. **(G)** Transwell invasion assay revealed the invasion of NSCLC cells. Data are expressed as mean ± SD. *p < 0.05, **p < 0.01 represents statistical difference.

The interaction among LINC00460, miR-769-5p, and EGFR has been identified in the functional and mechanical experiments. Furthermore, more assays are carried out to validate the biological roles. Pearson's correlation analysis indicated that LINC00460 was positively correlated with EGFR expression, and miR-769-5p was negatively correlated with EGFR expression (Fig. 4A, B). Western blots showed that EGFR expression was highly regulated in the gefitinib-resistant NSCLC cells (A549/GR) (Fig. 4C). Then, we also observed that EGFR protein expression was decreased in the transfection of both si-LINC00460 and miR-769-5p mimics, revealing the correlation between LINC00460, miR-769-5p, and EGFR (Fig. 4D, E). Chemotherapy-sensitive tests stated that the IC50 value of gefitinib in A549/GR cells was increased when cotransfected with the EGFR overexpression plasmids (Fig. 4F). Besides, the invasion of NSCLC cells was enhanced in the cotransfection with the EGFR overexpression plasmids (Fig. 4G). Therefore, these data state that EGFR enhances the role of LINC00460 in the gefitinib chemotherapy resistance of NSCLC cells.

## Discussion

In the current mainstream treatments for the human cancers, the combined surgical section and chemotherapy are the most commonly used therapeutic methods (Lin *et al.*, 2018). After the surgical excision, subsequent chemotherapy is administered to eliminate the residual tumor cells to prevent the relapse (Chen *et al.*, 2018). Gefitinib, belonging to the EGFR-TKIs, is specific for the advanced NSCLC patients with EGFR-sensitive mutations (Gonzalez-Larriba *et al.*, 2017; Mistry and Patil, 2017; Su *et al.*, 2017). In this study, we find that LINC00460 is ectopically expressed in the NSCLC tissue and cells, especially the gefitinib resistance.

The level of LINC00460 is significantly increased in the gefitinib-induced NSCLC cells compared with the parental cells. Moreover, functional experiments were carried out, indicating that LINC00460 could motivate and promote the gefitinib resistance and invasion of NSCLC cells. Thus, we identified the vital role of LINC00460 in the gefitinib chemotherapy resistance of NSCLC cells. Gefitinib resistance is a serious barrier to the NSCLC chemotherapy. Gefitinib is targeted to the patients with exon 19 deletion or L858R point mutation (Rawluk and Waller, 2018). It has been approved that gefitinib acts as the first-line treatment for NSCLC patients with EGFR-sensitive mutations. Gefitinib is found to be metabolized in liver. Gefitinib serves as a more suitable agent for treating advanced NSCLC patients with equal antitumor efficacy and fewer adverse effects (Zhang and Wei, 2018).

In the mechanical investigation, we find that LINC00460 inhibits EGFR protein expression. Moreover, the subcellular location of LINC00460 is established in the cytoplasm. The interesting findings spark the inspiration whether LINC00460 positively regulates EGFR expression through post-transcriptional control. Bioinformatics analysis confirms that miR-769-5p targets the 3′-UTR of LINC00460, validating the miRNA “sponge” of LINC00460. What's more, we also identified that EGFR acts as the functional protein of miR-769-5p. The LINC00460-miR-769-5p-EGFR axis was convincingly approved.

miR-769-5p has been confirmed to inhibit the lung tumorigenesis by silencing protein. For examples, TGFBR1 is identified as a direct target gene of miR-769-5p, and miR-769-5p exerts the tumor-repressive effects on NSCLC (Yang *et al.*, 2017). In this study, we discover that miR-769-5p targets EGFR and inhibits its expression. On the contrary, LINC00460 functions as the oncogenic RNA in the NSCLC cells. All the results support that LINC00460 promotes the gefitinib resistance of NSCLC cells by targeting EGFR through sponging miR-769-5p.

Recently, TKIs-based chemotherapy has been applied as a standard adjunctive treatment strategy in advanced NSCLC patients after surgical resection (Gainor *et al.*, 2017; Kong *et al.*, 2017; Prabhu and Devaraj, 2017). The research on lncRNA and the gefitinib resistance of NSCLC cells has been reported. For instance, lncRNA RHPN1-AS1 is downregulated in gefitinib-resistant patients, and NSCLC cell lines and RHPN1-AS1 knockdown promote the gefitinib resistance; however, RHPN1-AS1 upregulation activates the gefitinib-resistant NSCLC cells (Li *et al.*, 2018). Interestingly, RHPN1-AS1 exerts its effects through miR-299-3p/TNFSF12 pathway to modulate gefitinib resistance in NSCLC.

In this study, our research finds that the lncRNA LINC00460 is highly regulated in the gefitinib-resistant NSCLC cells. Just to make it more interesting, LINC00460 promotes the gefitinib resistance of NSCLC cells through sponging miR-769-5p, thereby promoting EGFR expression.

## Supplementary Material

Supplemental data
